# Abdominal Arterial Translation in Lower Lumbar Spine Level Due to Positional Change: A Clinical Survey Using Intraoperative Computed Tomography

**DOI:** 10.3390/jcm13071897

**Published:** 2024-03-25

**Authors:** Toru Asari, Kanichiro Wada, Eiji Sasaki, Gentaro Kumagai, Sunao Tanaka, Yasuyuki Ishibashi

**Affiliations:** Department of Orthopedic Surgery, Hirosaki University Graduate School of Medicine, Hirosaki 036-8562, Aomori, Japan

**Keywords:** complication of lumbar surgery, lumbar surgery with posterior approach, abdominal arterial position, intraoperative computed tomography, preoperative computed tomography, O-arm^®^

## Abstract

**Background**: Abdominal vascular injury, a fatal complication of lumbar disc surgery, should concern spine surgeons. This study aimed to compare the position of the abdominal arteries in the supine and prone positions and the factors involved. Thirty patients who underwent lumbar surgery by posterior approach were included. **Methods**: All patients underwent computed tomography (CT) preoperatively in the supine position and intraoperatively in the prone position. In the CT axial image, at the L4, L4/5 disc, L5, and L5/S1 disc level, we measured the shortest distance between the abdominal arteries and the vertebral body (SDA: shortest distance to the aorta), and the amount of abdominal arterial translation, defined as “SDA on intraoperative CT” minus “SDA on preoperative CT”. Additionally, the preoperative CT axial images were evaluated for the presence of aortic calcification. **Results**: No significant difference in SDA values based on patients’ positions was observed at each level. In males, the supine position brought the abdominal artery significantly closer to the spine at the left side of the L5/S level (*p* = 0.037), and, in cases of calcification of the abdominal artery, the abdominal artery was found to be closer to the spine at the left side of the L4/5 level (*p* = 0.026). **Conclusions**: It is important to confirm preoperative images correctly to prevent great vessel injuries in lumbar spine surgery using a posterior approach.

## 1. Introduction

The resection of the lumbar disc is necessary during herniotomy for lumbar disc herniation [1], as is posterior or transforaminal lumbar interbody fusion for lumbar spinal stenosis with instability and spondylolisthesis [2]. Complications of this so-called lumbar disc surgery have been reported, especially in patients with osteoporosis, including bony injury of the vertebral endplate [3,4], subsidence of the interbody cage [5,6], and cage migration into the retroperitoneal space [7].

Vascular injury at the ventral side of the lumbar vertebral body is a fatal complication of lumbar disc surgery that should concern spine surgeons. The incidence of vascular injuries during lumbar disc surgery has been reported to be 0.03–0.17%, with the most common site of occurrence at the L4/5 level and the second most common at the L5/S1 level, which together account for approximately 90% of all cases [8,9,10]. Types of vascular injuries include laceration, arteriovenous fistula (AVF), and pseudoaneurysm [10].

To prevent vascular injury, it is important for spine surgeons to understand the positional relationship between the lumbar spine and abdominal vessels when the patient is in the prone position under general anesthesia. However, to the best of our knowledge, there are no studies that report on the amount of vascular translation in the anterior–posterior direction when the patient is moved from the supine to the prone position under general anesthesia. Furthermore, a previous report analyzing the relationship between the location of the bifurcation in the cephalocaudal direction of the aorta and sex, age, calcification of the aorta, and body mass index (BMI) reported that older age, low BMI, and a history of smoking tended to move the bifurcation caudally. It has also been reported that individuals with calcium in any vascular bed had a lower bifurcation distance, a finding that was of marginal statistical significance [11]. However, it is unknown whether the age, sex, BMI, or the presence of aortic calcification affects the amount of vascular translation.

Therefore, the purpose of this study was to compare preoperative computed tomography (CT) images taken in the supine position with intraoperative CT images taken in the prone position during lumbar disc surgery to investigate the amount and direction of vascular translation due to positional change, and further to investigate the relationship between the amount of vascular translation and aortic calcification, age, sex, or BMI.

## 2. Materials and Methods

### 2.1. Participants

Thirty patients (10 men and 20 women) who underwent posterior lumbar interbody fusion between February 2018 and March 2021 were included in the study. All patients provided written informed consent before participating in the study. The study was performed in accordance with the ethical standards as laid down in the 1964 Declaration of Helsinki and its later amendments. All patients had lumbar spinal canal stenosis, and four of them also had lumbar spondylolisthesis. The exclusion criteria of this study were a history of abdominal vascular surgery or retroperitoneal organ surgery. In total, 17 patients underwent single-level interbody fusion and 13 underwent double-level interbody fusion. After induction of general anesthesia, the surgeons moved the patient from the supine to the prone position on the Jackson table (Mizuho OSI, Union City, CA, USA), and then the patient’s abdomen was suspended over the Jackson table to ensure it was not compressed.

### 2.2. Evaluation Method of Preoperative and Intraoperative CT Images

**Figure 1:**
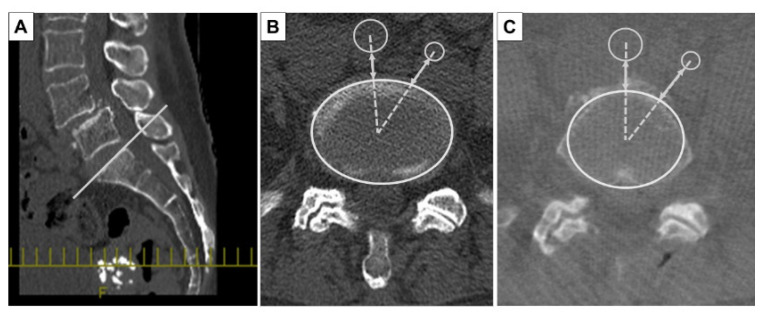
Measurement method of computed tomography (CT) images. Multi-planer reconstruction (MPR) Works software (GE Healthcare) was used to measure the shortest distance between the abdominal arteries and the vertebral body or intervertebral disc (SDA) at each level. When measuring the disc level, the MPR line was positioned in the sagittal plane at the cranial endplate of the caudal vertebral body (**A**), a line was drawn to connect the center point of the disc with the center point of the artery, and the shortest distance (two-way arrow) between the anterior margin of the disc and the artery wall margin was measured. Preoperative CT (**B**) and intraoperative CT (**C**) images were analyzed at each level precisely.

**Figure 2:**
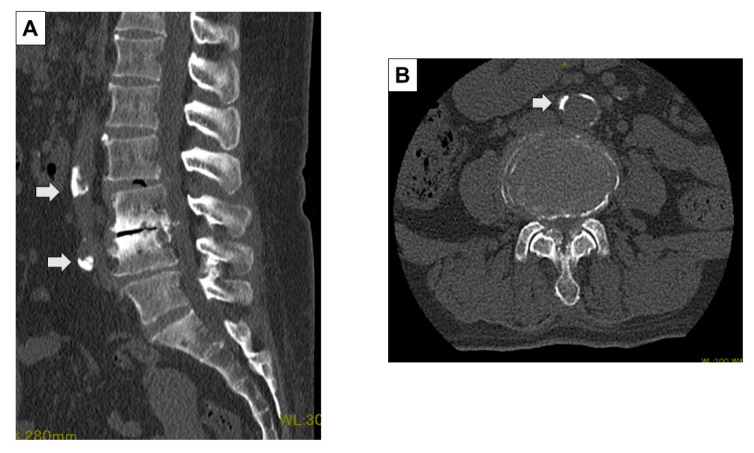
Evaluation of calcification using preoperative CT images. The presence of aortic calcification, and the bifurcation level to the common iliac artery were evaluated in the preoperative CT sagittal (**A**) and axial images (**B**).

All patients underwent preoperative and intraoperative CT. The preoperative CT (Discovery CT750 HD, Revolution CT, GE Healthcare, Waukesha, WI, USA, WL: 300, WW: 1200) was performed with the patient in the supine position. The intraoperative CT (O-arm^®^, Medtronic Sofamor Daneck, Memphis, TN, USA) was performed with the patient in the prone position when spine surgeons inserted pedicle screws into the vertebral bodies. The preoperative and intraoperative CT images were analyzed with multi-planer reconstruction (MPR) Works software (GE Healthcare, Waukesha, WI, USA) to measure each level precisely. In the CT axial image, at the L4 pedicle, L4/5 disc, L5 pedicle, and L5/S1 disc level, we measured the shortest distance between the abdominal arteries (aorta or common iliac artery, internal iliac artery) and the vertebral body or intervertebral disc (SDA: shortest distance to the aorta) on a line connecting the center of the abdominal arteries and the vertebral body or intervertebral disc (Figure 1) [12]. The amount of abdominal arterial translation (AAT) during surgery was defined as “SDA on intraoperative CT” minus “SDA on preoperative CT”. In addition, the presence of aortic calcification, and the bifurcation level to the common iliac artery were evaluated in the preoperative CT axial images (Figure 2).

### 2.3. Statistical Analysis

In all patients, the position of the abdominal artery at each level in the supine position and in the prone position were compared using the Wilcoxon signed-rank test. Spearman’s rank correlation coefficient was employed to correlate the amount of abdominal aortic migration with age and BMI. Patients were divided into two groups according to their sex or the presence of abdominal aortic calcification, and the amount of abdominal aortic migration was compared between the two groups using the Mann–Whitney U test. To analyze the relationship between AAT and the presence of calcification in the abdominal arteries, linear regression analysis was performed with AAT at the prone position as the dependent variable, and age, sex, BMI, diabetes, and the presence of calcification in the abdominal arteries at the L4 pedicle (L4P), L4/5 left, L4/5 right, L5 pedicle (L5P) left, L5P right, L5/S left, and L5/S right levels as independent variables. Values were expressed as mean ± standard deviation. Data input and analysis were performed using SPSS version 27.0 J (SPSS Inc., Chicago, IL, USA). A *p*-value < 0.05 was considered statistically significant.

## 3. Results

The mean age of the total 30 patients at the time of surgery was 65.3 ± 10.0 years (47–83 years), of which 20 (66.7%) were women, and the BMI was 26.7 ± 4.1 kg/m^2^. Divided into two groups according to the presence or absence of calcification of the abdominal aorta, the group without calcification consisted of 11 patients, with a mean age of 58.2 ± 7.9 (range: 47–72) years, 10 women (90.9%), and BMI of 29.0 ± 3.8 kg/m^2^. On the other hand, the group with calcification consisted of 19 patients, with a mean age of 69.5 ± 8.8 (range: 55–83) years, 10 women (52.6%), and BMI of 25.4 ± 3.9 kg/m^2^. The group with calcification was significantly older, had a smaller proportion of women, and had a lower BMI than the group without calcification (Table 1). With regard to vascular bifurcation, the branch from the abdominal aorta to the common iliac artery was located at the cranial side of the L4 pedicle in 1 case, between the L4 pedicle and L4/5 disc in 27 cases, and between the L4/5 disc and L5 pedicle in 2 cases. The right common iliac artery bifurcation was present from the L4 pedicle to the L4/5 disc in 1 case, between the L4/5 disc and the L5 pedicle in 1 case, between the L5 pedicle and the L5/S disc in 26 cases, and the remaining 2 cases did not branch even at the L5/S disc level.

Values and standard deviations are shown and compared by Mann–Whitney U test. Categorical values are compared by chi-square test.

The left common iliac artery bifurcation was present between the L5 pedicle and the L5/S disc in 24 cases, and the remaining 6 cases were not bifurcated even at the L5/S disc level.

**Figure 3:**
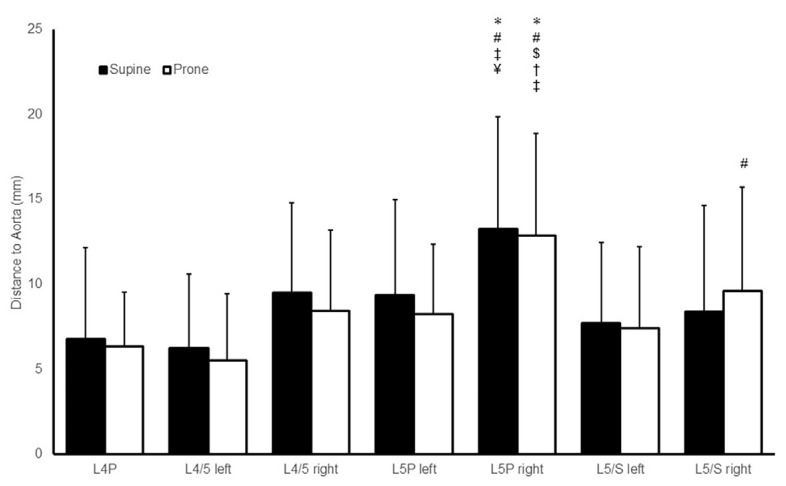
The shortest distance between the abdominal arteries and the vertebral body or intervertebral disc (SDA) at each level. Mean and standard deviations of the distances of the supine and prone positions are shown. The distances among seven points were compared by analysis of variance and the Tukey test. A *p*-value below 0.05 was considered significant in comparison with L4P (*), L4/5 left (#), L4/5 right ($), L5P left (†), L5/S left (‡), and L5/S right (¥).

In the 30 patients, the SDA at each level of preoperative CT were 6.7 ± 5.3 mm, 6.2 ± 4.3 mm, 9.5 ± 5.2 mm, 9.3 ± 5.6 mm, 13.2 ± 6.5 mm, 7.6 ± 4.7 mm, and 8.4 ± 6.2 mm at the L4P, L4/5 left, L4/5 right, L5P left, L5P right, L5/S left, and L5/S right levels, respectively. Conversely, the SDA at each level of intraoperative CT were 6.3 ± 3.1 mm, 5.4 ± 3.9 mm, 8.2 ± 4.7 mm, 8.2 ± 4.1 mm, 12.8 ± 5.9 mm, 7.4 ± 4.7, and 9.6 ± 6.0 mm at the L4P, L4/5 left, L4/5 right, L5P left, L5P right, L5/S left, and L5/S right levels, respectively. No significant difference in SDA values because of the patients’ position was observed at any level. The SDA at the L5P right level in the supine position was significantly higher than that of the L4P, L4/5 left, L5/S left, and L5/S right level. The SDA at the L5P right level in the prone position was significantly greater than all other SDA except for the L5/S right level (Figure 3).

Table 2 shows the results of the SDA measurements in the supine and prone positions at each level, separately with and without calcification of the abdominal arteries. Regardless of the presence or absence of calcification in the abdominal arteries, the SDA values were not significantly different between the supine and prone positions.

The results of the SDA measurements in the supine and prone positions for each level, separately for men and women, are shown in Table 3. Regarding these results, there was a significant difference in SDA between the supine and prone positions at the L5/SL level in men. There were no significant differences in SDA at any other level in men and at all levels in women between the supine and prone positions.

The correlations between age, sex, and BMI, respectively, and AAT were examined and no correlations were found with AAT, respectively. Linear regression analysis to examine the association between AAT and the presence of calcification in the abdominal arteries revealed that the presence of calcification was related to AAT at the L4/5 left level. At other levels, it was found that the presence of calcification was not associated with SDA in the prone position or AAT (Table 4).

## 4. Discussion

To the best of our knowledge, this study is the first to compare preoperative and intraoperative CT images to determine the direction in which the abdominal artery moves in relation to the spine depending on the body position. In a total of 30 cases studied, no significant difference in SDA values based on patients’ position was observed at each level. However, on the L5P right levels, both in the supine and prone positions, the abdominal artery was found to be positioned further away from the spine than at many other levels. When the patients were divided into those with and without calcification of the abdominal arteries, the SDA values were not significantly different between the supine and prone positions regardless of the presence or absence of calcification. Similarly, when men and women were examined separately, the SDA was significantly smaller in the prone position at the L5/S left level in men. Correlation analysis between age or BMI and AAT demonstrated no significant correlation. After adjusting for age, sex, and BMI, linear regression analysis revealed that the presence of abdominal artery calcification brought the abdominal artery significantly closer to the intervertebral disc at the L4/5 left level in the prone position.

When performing spine surgery, it is very important to know the location of the great vessels around the spinal column. There have been reports of great vessel injuries not only in the lumbar spine, but also in the cervical and thoracic spine in surgeries performed with the posterior approach. The incidence of vertebral artery injury through cervical spine surgery performed with the posterior approach has been reported to be 0.2–4.1% [13,14]. Internal carotid artery injury caused by upper cervical spine surgery, a serious complication, has been reported [15,16]. Furthermore, in the thoracic spine, there have been reports of postoperative iatrogenic perforation of the aorta [17] and late injury due to aortic wall erosion from the screw tip [18,19]. Therefore, great vessel injury is one of the most avoidable complications, regardless of the level of the spine, because it is a fatal complication. Hence, it is important to analyze how the great vessels translate because of positional change, and not just anatomical positioning.

Several reports have analyzed the location of the great vessels in spinal surgery patients using imaging. In the thoracic spine, Plataniotis et al. took CT scans of 200 patients in the supine, prone, and prone with padding positions and analyzed the positional relationship between the thoracic spine and the aorta in different positions [20]. They compared the shortest distance from the entry position of the pedicle screws to the aortic wall in the three positions and reported that the supine position was the closest and the prone position the furthest at all thoracic spine levels. Particularly at the T6 level, the aortic wall was located at the shortest distance from the typical screw trajectory. Therefore, they concluded that surgeons should be aware that standard supine CT evaluation represents a static technique, which can differ considerably from surgical reality.

In the lumbar spine, Ganesan et al. previously performed MRI imaging in the supine, prone, and prone with bolsters positions on seven patients and measured the distance between the aorta and the anterior margin of the intervertebral disc at the L4/5 level [21]. They reported that placing bolsters under the patients in the prone position did not increase the distances between the disc spaces and the arteries, and the distance to the aorta at the L4/5 level in all positions was an average of 4.3 mm, and that the distance did not change with position. Here, the patient was also prone on the Jackson table under general anesthesia, and although we reviewed a larger number of cases, we did not find any significant changes in SDA depending on the body position.

The Jackson table may have also influenced our results. Ni et al. reported that during lumbar spine surgery, the Jackson table increased the oxygenation index and decreased intraperitoneal pressure, unlike other surgical tables, even when the patient was in the prone position [22]. Although we did not measure intraperitoneal pressure in our research, considering that vascular migration was not affected by positional change, this may indicate that intraperitoneal pressure was relevant.

Since our results show that SDA did not significantly change with position, it is important to confirm preoperative images correctly to prevent great vessel injuries in lumbar spine surgery with a posterior approach. Adjusting for age, sex, and BMI, linear regression analysis revealed that in the presence of arterial calcification, the abdominal artery is significantly closer in the prone position at the L4/5 left level. Therefore, in cases with calcification of the abdominal artery, arterial injury should be kept in mind during the L4/5 left disc manipulation technique. Reports of great vessel injury at the L4/5 level should be given special attention, as this is also the level most frequently reported in previous articles to be associated with arterial injuries.

This study has several limitations. Firstly, the location of the abdominal arteries was evaluated using plain CT. Plain CT can be used to evaluate the abdominal arteries, but contrast-enhanced CT provides a more accurate evaluation of the arteries, and the use of contrast-enhanced CT is ideal. However, it is difficult to perform contrast-enhanced CT as an intraoperative CT, therefore only plain CT was used. Secondly, we did not evaluate the abdominal veins. Since many of the cases reported calcification of the abdominal arteries, it was determined that the arteries could be easily evaluated; however, the veins could not be accurately evaluated and therefore the arteries were analyzed. Thirdly, the participants in this study were people who had undergone lumbar spine surgery, and healthy or young people were not included. In practice, it would be ethically difficult to obtain data from healthy individuals under general anesthesia and in the prone position for CT imaging. It is also difficult to obtain data from such subjects from the standpoint of medical radiation exposure. Fourthly, the sample size was small. Future analysis with a larger sample size may reveal new factors related to AAT.

## 5. Conclusions

SDA did not change significantly when the location of the abdominal arteries was examined in the supine and prone positions in lumbar spine surgery cases with a posterior approach. In male patients, the supine position brought the abdominal artery significantly closer to the spine at the L5/S left level, and in cases with calcification of the abdominal artery, the abdominal artery was closer to the spine at the L4/5 left level. Therefore, it is important to confirm preoperative images correctly to prevent great vessel injuries in lumbar spine surgery using a posterior approach. Increasing the sample size and further analysis of comorbidity details may reveal new factors associated with AAT in the future.

## Figures and Tables

**Table 1:** Patient demographics with or without calcification of the aorta.

	Total	Calcification (−)	Calcification (+)	*p*-Value
Sample number	30	11	19	
Age	65.3 ± 10.0	58.2 ± 7.9	69.5 ± 8.8	0.002
Female	20 (66.7%)	10 (90.9%)	10 (52.6%)	0.049
Height	155.9 ± 7.8	154.7 ± 5.2	156.7 ± 9.1	0.672
Body weight	65.1 ± 11.7	69.5 ± 10.0	62.6 ± 12.2	0.103
Body mass index	26.7 ± 4.1	29.0 ± 3.8	25.4 ± 3.9	0.016

**Table 2:** No remarkable arterial translation in positional change regardless of calcification.

	Calcification (−)				Calcification (+)			
	Supine	Prone	*p*-Value	AAT	Supine	Prone	*p*-Value	AAT
L4P	7.5 ± 8.4	5.5 ± 3.8	0.248	−2.0 ± 5.3	6.4 ± 2.6	6.8 ± 2.7	0.355	0.4 ± 1.8
L4/5 left	4.9 ± 5.0	5.2 ± 4.9	0.248	0.3 ± 2.4	7.0 ± 4.0	5.7 ± 3.5	0.334	−1.3 ± 3.5
L4/5 right	9.7 ± 6.0	9.3 ± 5.2	0.799	−0.4 ± 1.9	9.4 ± 5.0	8.0 ± 4.6	0.133	−1.4 ± 3.7
L5P left	7.7 ± 7.4	6.4 ± 3.7	0.929	−1.3 ± 5.7	10.3 ± 4.3	9.3 ± 4.1	0.107	−1.0 ± 3.7
L5P right	11.5 ± 7.1	10.7 ± 5.6	0.657	−0.8 ± 7.0	14.3 ± 6.2	14.2 ± 5.9	0.601	−0.1 ± 4.3
L5/S left	5.7 ± 3.5	7.5 ± 6.2	0.248	1.7 ± 4.1	8.8 ± 5.1	7.4 ± 3.9	0.159	−1.4 ± 3.8
L5/S right	7.6 ± 5.7	8.5 ± 5.3	0.213	1.0 ± 3.1	8.9 ± 6.7	10.3 ± 6.5	0.445	1.4 ± 6.5

Means and standard deviations of the shortest distance between the abdominal arteries and the vertebral body or intervertebral disc examined at supine and prone position. The values between supine and prone were compared by Wilcoxon signed-rank test. AAT, abdominal arterial translation.

**Table 3:** The shortest distance between the abdominal arteries and the vertebral body or intervertebral disc in supine and prone positions by sex.

	Men			Women		
	Supine	Prone	*p*-Value	Supine	Prone	*p*-Value
L4P	7.9 ± 2.6	8.0 ± 2.6	0.799	6.2 ± 6.3	5.5 ± 3.2	0.940
L4/5 left	8.0 ± 4.4	6.8 ± 3.8	0.646	5.3 ± 4.2	4.9 ± 4.0	0.940
L4/5 right	11.0 ± 5.0	9.4 ± 4.8	0.203	8.7 ± 5.4	7.9 ± 4.8	0.231
L5P left	10.3 ± 5.7	10.0 ± 4.8	0.878	8.9 ± 5.7	7.4 ± 3.6	0.156
L5P right	15.7 ± 7.4	17.3 ± 6.1	0.575	12.0 ± 5.9	10.7 ± 4.7	0.502
L5/S left	8.6 ± 6.4	5.6 ± 3.4	0.037	7.2 ± 3.8	8.3 ± 5.2	0.145
L5/S right	10.0 ± 8.3	12.0 ± 7.7	0.575	7.6 ± 5.0	8.4 ± 4.9	0.332

**Table 4:** Relationship between the amount of abdominal arterial translation and aortic calcification.

Calcification of Aorta At	β	*p*-Value	R^2^
L4P	0.215	0.379	0.109
L4/5 left	−0.521	0.026	0.325
L4/5 right	−0.156	0.548	0.072
L5P left	−0.247	0.281	0.232
L5P right	−0.29	0.157	0.397
L5/S left	−0.253	0.203	0.426
L5/S right	−0.020	0.933	0.172

Linear regression analysis was performed with translation of aorta at L4P, L4/5 left, L4/5 right, L5P left, L5P right, L5/S left, and L5/S right as dependent variables, respectively. Each model was adjusted by age, sex, body mass index, and diabetes as confounders for elasticity of the aorta.

## Data Availability

The datasets used and/or analyzed during the current study are available from the corresponding author upon reasonable request.
